# Pressure-Controlled Ventilation-Volume Guaranteed Mode Combined with an Open-Lung Approach Improves Lung Mechanics, Oxygenation Parameters, and the Inflammatory Response during One-Lung Ventilation: A Randomized Controlled Trial

**DOI:** 10.1155/2020/1403053

**Published:** 2020-04-29

**Authors:** Jianli Li, Baogui Cai, Dongdong Yu, Meinv Liu, Xiaoqian Wu, Junfang Rong

**Affiliations:** Department of Anesthesiology, Hebei General Hospital, Shijiazhuang 050051, China

## Abstract

We evaluated the effectiveness of pressure-controlled ventilation-volume guaranteed (PCV-VG) mode combined with open-lung approach (OLA) in patients during one-lung ventilation (OLV). First, 176 patients undergoing thoracoscopic surgery were allocated randomly to four groups: PCV+OLA (45 cases, PCV-VG mode plus OLA involving application of individualized positive end-expiratory pressure (PEEP) after a recruitment maneuver), PCV (44 cases, PCV-VG mode plus standard lung-protective ventilation with fixed PEEP of 5 cmH_2_O), VCV+OLA (45 cases, volume-controlled ventilation (VCV) plus OLA), and VCV (42 cases, VCV plus standard lung-protective ventilation). Mean airway pressure (*P*_mean_), dynamic compliance (Cdyn), PaO_2_/FiO_2_ ratio, intrapulmonary shunt ratio (Qs/Qt), dead space fraction (*V*_D_/*V*_T_), and plasma concentration of neutrophil elastase were obtained to assess the effects of four lung-protective ventilation strategies. At 45 min after OLV, the median (interquartile range (IQR)) *P*_mean_ was higher in the PCV+OLA group (13.00 (12.00, 13.00) cmH_2_O) and the VCV+OLA group (12.00 (12.00, 14.00) cmH_2_O) than in the PCV group (11.00 (10.00, 12.00) cmH_2_O) and the VCV group (11.00 (10.00, 12.00) cmH_2_O) (*P* < 0.05); the median (IQR) Cdyn was higher in the PCV+OLA group (27.00 (24.00, 32.00) mL/cmH_2_O) and the VCV+OLA group (27.00 (22.00, 30.00) mL/cmH_2_O) than in the PCV group (23.00 (21.00, 25.00) mL/cmH_2_O) and the VCV group (20.00 (18.75, 21.00) mL/cmH_2_O) (*P* < 0.05); the median (IQR) Qs/Qt in the PCV+OLA group (0.17 (0.16, 0.19)) was significantly lower than that in the PCV group (0.19 (0.18, 0.20)) and the VCV group (0.19 (0.17, 0.20)) (*P* < 0.05); *V*_D_/*V*_T_ was lower in the PCV+OLA group (0.18 ± 0.05) and the VCV+OLA group (0.19 ± 0.07) than in the PCV group (0.21 ± 0.07) and the VCV group (0.22 ± 0.06) (*P* < 0.05). The concentration of neutrophil elastase was lower in the PCV+OLA group than in the PCV, VCV+OLA, and VCV groups at total-lung ventilation 10 min after OLV (162.47 ± 25.71, 198.58 ± 41.99, 200.84 ± 22.17, and 286.95 ± 21.10 ng/mL, resp.) (*P* < 0.05). In conclusion, PCV-VG mode combined with an OLA strategy leads to favorable effects upon lung mechanics, oxygenation parameters, and the inflammatory response during OLV.

## 1. Introduction

One-lung ventilation (OLV) has been used routinely in thoracic surgery to provide an optimal visual field for a surgical procedure on a collapsed lung. Unfortunately, this approach creates a “shunt-like” effect through the nondependent lung and results in hypoxemia [1]. Furthermore, OLV and surgical trauma are accompanied by the release of excessive amounts of inflammatory mediators and neutrophil elastase, which lead to pulmonary infection and systemic inflammatory response syndrome [2, 3].

Lung-protective ventilation, consisting a tidal volume (*V*_T_) of 5–6 mL/kg of predicted body weight (PBW) and positive end-expiratory pressure (PEEP) of 5 cmH_2_O with an alveoli recruitment maneuver at 20 cmH_2_O for 15–20 s, can reduce the risk of ventilator-induced lung injury [4]. However, except for low *V*_T_, the advantages of appropriate PEEP with or without an alveoli recruitment maneuver have not been established exactly [5, 6]. Alternatively, an open-lung approach (OLA), in which the individualized PEEP is determined by PEEP titration after an alveoli recruitment maneuver, can contribute to favorable physiologic effects [7, 8].

The literature regarding the ideal ventilation mode on pulmonary outcomes for OLV is controversial [9–11]. Volume-controlled ventilation (VCV) ensures a stable minute ventilation volume, but this mode generates a high airway pressure with subsequent volutrauma and barotrauma and leads to uneven distribution of gas in the lungs. Pressure-controlled ventilation (PCV) offers the benefits of lower airway pressure with a decelerating flow pattern, but it can provoke lung injury due to a tractive force on alveoli [12]. Pressure-controlled ventilation-volume guaranteed (PCV-VG) mode is a relatively innovative ventilation model introduced to the operating theatre. In PCV-VG mode, initially a preset *V*_T_ is transmitted at a lower airway pressure by a decelerating flow. Upon calculation of a patient's pulmonary compliance and inspiratory pressure, the ventilator automatically adjusts the airway pressure of the next breath according to the previous breath's measured exhaled *V*_T_ [13, 14]. Recently, different results by comparing the efficacy of PCV-VG over other modes were published [9, 14]. Nevertheless, lung-protective ventilation applied to PCV-VG mode has not been studied deeply, and whether PCV-VG combined with an OLA is superior to VCV plus standard lung-protective ventilation during OLV is not known.

We carried out the study to explore the benefits of PCV-VG mode in combination with an OLA on lung mechanics, oxygenation parameters, and the inflammatory response during thoracic surgery.

## 2. Materials and Methods

### 2.1. Study Population

The ethics committee of Hebei General Hospital (Hebei, China) approved the study protocol. Each participant (or family member) provided written informed consent. The study was registered with the Chinese Clinical Trial Registry (ChiCTR1900020895).

The study enrolled patients with ASA physical status I–III scheduled for elective thoracoscopic surgery requiring OLV. Patients were excluded if they met any of the following criteria: age < 18 years, body mass index ≥ 35 kg/m^2^, pneumothorax or giant bullae, chronic lung disease or pulmonary infection within one month of study initiation, previous thoracic surgery, and contraindication to PEEP (high intracranial pressure, hypovolemic shock, or right-heart failure). Dropout criteria were a change in type of surgical procedure to thoracotomy, intraoperative bleeding ≥ 500 mL, and OLV duration < 45 min.

### 2.2. Randomization

Participants were assigned to one of four lung-protective ventilation strategies using a computer-generated randomization sequence, with an allocation of 1 : 1 : 1 : 1.

### 2.3. Anesthesia and Surgery

After placement of monitors, anesthesia induction and endobronchial intubation were achieved with 0.3 mg/kg etomidate, 0.3 *μ*g/kg sufentanil, and 1.0 mg/kg rocuronium. The location of the left- or right-sided double-lumen tube (DLT) was regulated by a fiberoptic bronchoscope in supine and lateral positions. Anesthesia was maintained with sevoflurane, remifentanil, and rocuronium. Sevoflurane was titrated to keep the bispectral index between 40 and 60. Lactated Ringer's solution was infused continuously at 3–5 mL/kg/h. All patients received patient-controlled intravenous analgesia after surgery. And patients were treated in the thoracic surgery intensive care unit (ICU) after surgery.

### 2.4. General Ventilator Strategy

After endobronchial intubation, all patients in the four groups were ventilated with an anesthesia ventilator (Avance CS^2^ Pro; GE Healthcare, Piscataway, NJ, USA).

Before OLV, all participants were set the same ventilation parameters, consisting of a fraction of inspired oxygen (FiO_2_) of 1.0, *V*_T_ of 8 mL/kg PBW, and an initial PEEP of 5 cmH_2_O (which was maintained in the PCV group and the VCV group throughout the whole procedure).

During OLV, all individuals received a *V*_T_ of 5–6 mL/kg PBW with FiO_2_ of 0.8. The inspiratory : expiratory (I : E) ratio was 1 : 2, and ventilation frequency was adjusted to maintain end-tidal carbon dioxide partial pressure (P_ET_CO_2_) between 35 and 45 mmHg. The maximal acceptable peak airway pressure (*P*_peak_) was set at 30 cmH_2_O, and if *P*_peak_ was exceeded, VCV was switched to PCV.

### 2.5. Alveoli Recruitment Maneuver and Decremental PEEP Trial

OLV was initiated after rechecking the correct position of the DLT. The dependent lung had a standard alveoli recruitment maneuver. The ventilation mode was changed from VCV to PCV with a driving pressure of 20 cmH_2_O and respiratory rate of 15 breaths per minute, PEEP of 5 cmH_2_O, I : E of 1 : 1, and FiO_2_ of 1.0. PEEP was increased at a step size of 5 cmH_2_O, and 10 breaths were maintained at each step (5, 10, 15, and 20 cmH_2_O) until recruitment of opening pressure up to 40 cmH_2_O (20 cmH_2_O PEEP and 20 cmH_2_O of driving pressure) was applied for 20 breaths.

If the hemodynamics were unstable during the alveoli recruitment maneuver phase (a decrease in mean arterial pressure (MAP) > 30%), the alveoli recruitment maneuver was interrupted and vasoactive drugs given; after hemodynamic stability, a new alveoli recruitment maneuver was implemented.

**Figure 1:**
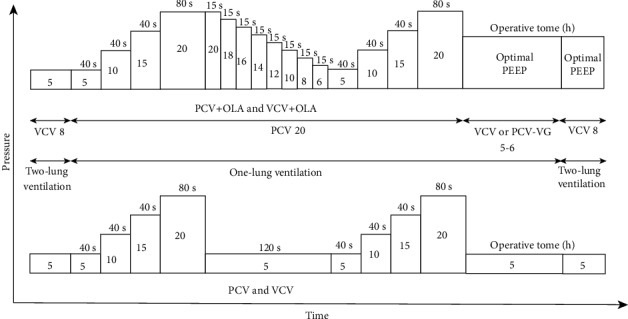
PCV 20: pressure-controlled ventilation mode with 20 cmH_2_O; VCV 8: volume-controlled ventilation mode with tidal volume set to 8 mL/kg; VCV or PCV-VG 5-6: volume-controlled ventilation mode or pressure-controlled ventilation-volume guaranteed mode with tidal volume set to 5 to 6 mL/kg.

After the first alveoli recruitment maneuver had been accomplished, individualized PEEP was titrated through a trial with decreased PEEP. PEEP was reduced in steps of 2 cmH_2_O with each PEEP level (20, 18, 16, 14, 12, 10, 8, and 6 cmH_2_O) and held for 15 s until the greatest dynamic compliance (Cdyn) was produced, which was considered to be the individualized or optimal PEEP. Then, a new alveoli recruitment maneuver was carried out as described above (Figure 1).

### 2.6. Specific Intraoperative Ventilatory Management

In the PCV+OLA group, after the second alveoli recruitment maneuver, the ventilation mode was changed to PCV-VG during OLV, and the optimal PEEP was established and maintained throughout the whole study period.

In the VCV+OLA group, patients received the same procedures (alveoli recruitment maneuver and trial of decreased PEEP), the ventilation mode was switched to VCV during OLV, and the optimal PEEP maintained throughout the whole study period.

In the PCV and VCV groups, the same procedure (alveoli recruitment maneuver) was followed except for the PEEP titration. Patients received PCV-VG or VCV plus fixed PEEP (5 cmH_2_O) during OLV, respectively.

It is worth noting that alveoli recruitment maneuver was performed after OLV without PEEP titration. The ventilation mode was changed to VCV, and the PEEP value of each group was consistent with that during OLV.

### 2.7. Measurements

Studied variables were collected at three time points: (i) T_1_: total-lung ventilation 10 min after intubation; (ii) T_2_: OLV for 45 min; and (iii) T_3_: total-lung ventilation 10 min after OLV.

The studied endpoints were partial pressure of arterial carbon dioxide (PaCO_2_), pH, *V*_T_, PEEP, mean airway pressure (*P*_mean_), Cdyn, *P*_peak_, dead space fraction (*V*_D_/*V*_T_), intrapulmonary shunt ratio (Qs/Qt), arterial partial pressure of oxygen/fraction of inspired oxygen (PaO_2_/FiO_2_ ratio), and the plasma concentration of neutrophil elastase.

Parameters were calculated using the following equations:

(1) $$\begin{array}{c} V_{D}/V_{T}=\left(PaCO_{2}-P_{ET}CO_{2}\right)/PaCO_{2}, \end{array}$$

(2) $$\begin{array}{c} Qs/Qt=\left(PA‐aDO_{2}\times 0.0031\right)÷\left(PA‐aDO_{2}\times 0.0031+5\right), \end{array}$$

whereby PA‐aDO_2_ = [FiO_2_ × (*P*_B_ − *P*_H2O_)] − (PaCO_2_/*R*) − PaO_2_.

PA-aDO_2_ is the alveolar-arterial oxygen difference; *P*_B_ is the barometric pressure (760 mmHg); *P*_H2O_ is the vapor pressure of water (47 mmHg); *R* is the respiratory quotient (0.8).

The plasma concentration of neutrophil elastase was measured by enzyme-linked immunosorbent assays at T_1_ and T_3_.

Postoperative endpoints in the four groups were recorded: prevalence of pneumonia, atelectasis, and acute respiratory failure; duration of ICU stay; and duration of hospital stay after surgery.

## 3. Statistical Analyses

The sample size for our study was determined according to a pilot study, with an *α* level of 0.05, power of 0.8, and effect size of 0.3. Assuming a dropout of 30% of cases, 200 patients (50 patients per group) were included in each group.

Statistical data were analyzed using SPSS 22.0 (IBM, Armonk, NY, USA). The Shapiro-Wilk test was used for data with a normal distribution. Continuous variables are given as the mean ± standard deviation (SD) or median (interquartile range (IQR)). Categorical variables are described as numbers. Categorical data were analyzed using the chi-squared test. Data with a normal distribution were compared among the four groups using one-way ANOVA with LSD-t as the *post hoc* test. The Kruskal-Wallis test was used to compare multiple groups on continuous variables with a nonnormal distribution. *P* < 0.05 (two-sided) was considered significant for all tests.

## 4. Results

### 4.1. Demographic and Intraoperative Characteristics of Patients

**Figure 2:**
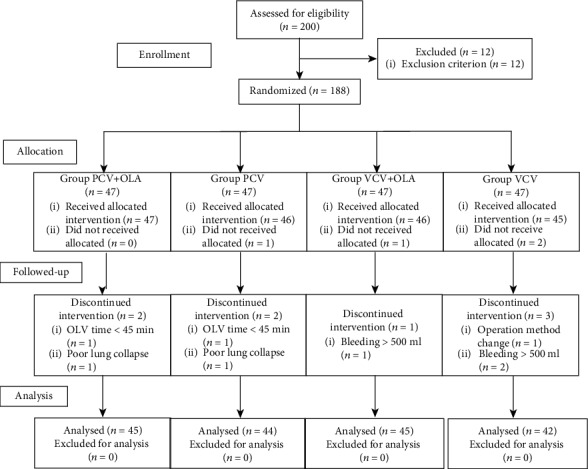
The study flow diagram.

Initially, 200 patients were assessed for eligibility, and 176 patients completed the study (Figure 2). The four groups were balanced in terms of demographics and data relating to the surgical procedure (*P* > 0.05) (Tables 1 and 2).

### 4.2. Mechanics of the Respiratory System

Compared with the PCV and VCV groups (both 5.00 cmH_2_O), the median (IQR) PEEP was higher in the PCV+OLA group (8.00 (8.00, 10.00) cmH_2_O) and the VCV+OLA group (10.00 (8.00, 12.00) cmH_2_O) (*P* < 0.001).

At T_2_, the median (IQR) *P*_mean_ was higher in the PCV+OLA group (13.00 (12.00, 13.00) cmH_2_O) and the VCV+OLA group (12.00 (12.00, 14.00) cmH_2_O) than in the PCV and VCV groups (both 11.00 (10.00, 12.00) cmH_2_O) (*P* < 0.05). The median (IQR) Cdyn was higher in the PCV+OLA group (27.00 (24.00, 32.00) mL/cmH_2_O) and the VCV+OLA group (27 (22.00, 30.00) mL/cmH_2_O) than in the PCV group (23.00 (21.00, 25.00) mL/cmH_2_O) and the VCV group (20.00 (18.75, 21.00) mL/cmH_2_O) at T_2_ (*P* < 0.05). At T_2_, *P*_peak_ showed no difference in the PCV+OLA and PCV groups (*P* = 0.320) or the VCV+OLA and VCV groups (*P* = 0.856) (Table 3).

### 4.3. Variables in Ventilation Efficiency

At T_2_, the median (IQR) Qs/Qt in the PCV+OLA group (0.17 (0.16, 0.19)) was significantly lower than that in the PCV group (0.19 (0.18, 0.20)) and the VCV group (0.19 (0.17, 0.20)) (*P* < 0.05). *V*_D_/*V*_T_ was lower in the PCV+OLA group (0.18 ± 0.05) and the VCV+OLA group (0.19 ± 0.07) than that in the PCV group (0.21 ± 0.07) and the VCV group (0.22 ± 0.06) at T_2_ (*P* < 0.05). Compared with the VCV group, the PaO_2_/FiO_2_ ratio increased in the PCV+OLA and VCV+OLA groups (median (IQR) 173.75 (138.13, 221.87) *vs.* 134.38 (106.25, 180.63); 166.25 (146.25, 200.63) *vs.* 134.38 (106.25, 180.63), *P* < 0.05) (Table 3).

### 4.4. Plasma Concentration of Neutrophil Elastase

**Figure 3:**
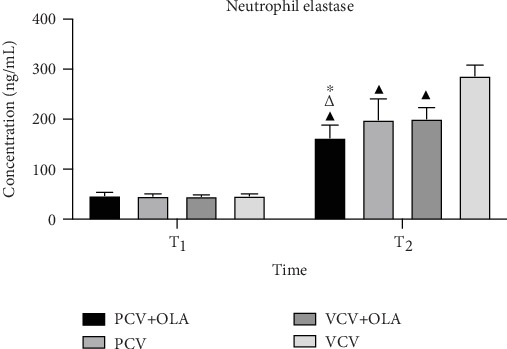
The plasma concentration of neutrophil elastase. Data are expressed as mean ± standard deviation (SD). ^∗^*P* < 0.05 versus PCV, ^△^*P* < 0.05 versus VCV+OLA, and ^▲^*P* < 0.05 versus VCV.

There was no significant difference in the plasma neutrophil elastase level among the four groups at T_1_ (*P* > 0.05). The plasma concentration of neutrophil elastase was lower in the PCV+OLA group than that in the PCV, VCV+OLA, and VCV groups at T_3_ (162.47 ± 25.71, 198.58 ± 41.99, 200.84 ± 22.17, and 286.95 ± 21.10, ng/mL, resp.) (*P* < 0.05) (Figure 3).

### 4.5. Other Clinical Endpoints

The duration of ICU stay in the PCV+OLA group was shorter compared with that in PCV, VCV+OLA, and VCV groups (median (IQR) 32.00 (25.00, 37.00), 39.75 (32.88, 43.00), 39.50 (27.00, 43.50), and 39.60 (24.88, 43.70) h, resp.) (*P* < 0.05). There were no significant differences in the duration of hospital stay and the prevalence of pneumonia, atelectasis, and acute respiratory failure in the four groups after surgery (*P* > 0.05) (Table 4).

## 5. Discussion

The randomized controlled trial revealed that the ventilation strategy of PCV-VG plus OLA during OLV leads to preferable levels of Cdyn, PaO_2_/FiO_2_ ratio, and appropriate levels of *P*_mean_, *V*_D_/*V*_T_, Qs/Qt, and neutrophil elastase. Additionally, the duration of ICU stay was shorter in the PCV+OLA group compared with the other three groups. These results suggest that the ventilation strategy of PCV-VG combined with OLA during OLV is beneficial for patients undergoing thoracic surgery.

Patients undergoing OLV are susceptible to hypoxemia due to shunting of blood or imbalance of ventilation and pulmonary perfusion. And the practice of OLV is an independent hazard factor for postoperative pulmonary complications (PPCs), as a result of direct surgical trauma of the nonventilated lung, exposed to high strain and nonphysiologic *V*_T_ of the ventilated lung [15, 16]. Multiple mechanisms can cause lung tissue damage and inflammatory cytokine release, which often ahead of pneumonia and systemic inflammatory response syndrome ultimately affect the clinical prognosis of patients undergoing thoracic surgery [17, 18].

Usually, the lung-protective ventilation strategy, which has taken low *V*_T_ as the core, is accepted by anesthesiologists as an effective way to alleviate ventilator-induced lung injury [19]. However, the application of a low *V*_T_ without sufficient PEEP may be relevant to cyclic alveolar derecruitment with consequent promote atelectrauma [6]. PEEP can prevent atelectasis effectively, but the optimal PEEP level is not known. Pereira et al. observed that PEEP varied markedly among individuals and stated that intraoperative individualized PEEP settings could lower the risk of postoperative atelectasis while improving driving pressure and oxygenation [20]. On the contrary, a study showed that different perioperative OLA with individualized PEEP in major abdominal surgery did not reduce the prevalence of postoperative complications when compared with conventional lung-protective ventilation strategy [21]. Most protective ventilation strategies (including the OLA) were undertaken under VCV mode, which could pose a potential risk of high airway pressure and trigger pulmonary inflammatory response compared with PCV or PCV-VG [10, 22]. The PCV-VG mode has features of PCV and VCV, which creates lower airway pressure than that seen with VCV accompanied with improvement in oxygenation and reduction in the pulmonary shunt [1, 13]. However, Byun and colleagues indicated that the application of *V*_T_ of 6 mL/kg with zero PEEP under PCV-VG led to a high risk of intraoperative hypoxemia [23]. Moreover, many studies found that compared with VCV, PCV-VG did not significantly improve intraoperative oxygenation [24, 25]. Hence, we designed this study to explore the benefits of PCV-VG along with an individualized OLA strategy in patients undergoing lung surgery with OLV.

The results of the present study are consistent with our expectation that using an OLA with individualized PEEP under PCV-VG mode can improve pulmonary gas exchange and lung mechanics as well as hemodynamic stability during OLV. Nevertheless, it is useful to note that during OLV, routinely measured airway pressure does not reflect the bronchial pressure, and the decrease in *P*_peak_ under PCV mode is probably not clinically relevant when measured in the bronchus of the ventilated lung [26]. Therefore, we assumed that the difference in *P*_peak_ in this study is meaningless due to the ventilation mode-related difference in end-inspiratory flow, as well as the resistance of the tracheal tube [27]. Unlike *P*_peak_, previous studies have demonstrated that *P*_mean_ closely reflects mean alveolar pressure and correlates with alveolar ventilation, arterial oxygenation, hemodynamic performance, and barotrauma under conditions of passive inflation [28, 29]. In this study, *P*_mean_ and Cdyn in the PCV+OLA and VCV+OLA groups were similar, and both were higher than those in the PCV and VCV groups. The reason may be related to the application of higher individualized PEEP. Indeed, others have shown that *P*_mean_ was significantly higher in the PCV+PEEP group compared with the VCV+zero PEEP and PCV+zero PEEP groups [24, 30]. Furthermore, it usually requires the application of extrinsic PEEP by increasing *P*_mean_ during OLV to prevent hypoventilation and atelectasis caused by low *V*_T_ [31–33]. As a matter of fact, an abnormally high *P*_mean_ may incur the impairment of pulmonary circulation and hemodynamic stability [34]. However, there was no difference in hemodynamics among the four groups, probably because the higher *P*_mean_ in the PCV+OLA and VCV+OLA groups did not substantially affect hemodynamic stability during OLV. On the other hand, excessively high *P*_mean_, especially mean alveolar pressure, increases pulmonary vascular resistance and makes blood flow towards the nonventilated lung, thus leading to the disturbance of Qs/Qt [29, 35, 36]. Nevertheless, the Qs/Qt in the PCV+OLA group was superior to that in the other groups during OLV, suggesting that acceptable *P*_mean_ recruits alveoli and tends to keep them properly open.

The higher PaO_2_/FiO_2_ ratio and lower *V*_D_/*V*_T_ of the PCV+OLA and VCV+OLA groups during OLV may have been owing to the impact of the OLA and reasonable *P*_mean_ on oxygenation and the prevention of atelectasis. We assumed that the most suitable PEEP might more effectively maintain the advantages of an alveoli recruitment maneuver with regard to ventilation efficiency under the automatic adjustment of PCV-VG mode. Unlike the constant flow pattern observed in VCV, the pattern of inspiratory flow in PCV-VG mode is a deceleration type with high initial flow velocity, which results in compliant alveolar expansion and improves ventilation-perfusion matching [37]. Therefore, the PCV+OLA ventilation strategy integrated the merits of PCV-VG and OLA to improve both gas exchange and oxygenation accordingly. Another explanation was that FiO_2_ of 0.8 used during OLV could decrease the number of areas suffering from atelectasis and, consequently, lead to lowering Qs/Qt [35, 38]. However, some studies showed that compared with VCV, no benefits were found in PCV about the length of hospital stay and PaO_2_/FiO_2_ ratio [26, 39]. Consistent with the negative results of these studies, the results of respiratory mechanics and ventilation efficiency in the PCV group and the VCV group were similar; the reason may be that except for the different ventilator modes, the other treatment measures were the same in these two groups.

The release of pulmonary inflammatory mediators and their cascade reaction during OLV are the major mechanisms resulting in acute lung injury [16]. Neutrophil elastase participates in and initiates acute lung injury by injuring capillary endothelial cells and alveolar epithelial cells, as well as digesting and degrading the extracellular matrix and epithelial junctions [40]. A recent study showed that compared with VCV, PCV-VG could decrease the release of neutrophil elastase and minimize inflammatory reaction to reduce lung injury in patients undergoing OLV [22]. Fernandez-Bustamante et al. proposed that plasma neutrophil elastase might indicate atelectrauma in patients with short-term mechanical ventilation [41]. We found that the plasma concentration of neutrophil elastase was decreased considerably in the PCV+OLA group compared with the PCV, VCV, and VCV+OLA groups after OLV, demonstrating that the strategy of PCV-VG plus OLA had an important influence on alleviating ventilator-induced lung injury.

Unfortunately, except for the shortening of the duration of ICU stay, the prevalence of other postoperative endpoints did not decrease. Whether the strategy of PCV-VG plus OLA can impact upon postoperative duration of hospital stay, total duration of hospital stay, or the incidence of PPCs requires further study at multiple centers.

Our study had two main limitations. First, our study was not blinded, so biases are inevitable. Second, we only observed changes in heart rate and MAP. We intend to determine the effect of PCV-VG plus OLA during OLV on hemodynamic variables by measuring central venous pressure and cardiac output.

## 6. Conclusion

The ventilation strategy of PCV-VG combined with open-lung approach during OLV was associated with favorable effects upon intraoperative respiratory mechanics, oxygenation parameters, and the inflammatory reaction. This ventilation strategy may be a feasible alternative ventilation method in patients undergoing thoracic surgery.

## Figures and Tables

**Table 1:** Patients' characteristics.

	PCV+OLA (*n* = 45)	PCV (*n* = 44)	VCV+OLA (*n* = 45)	VCV (*n* = 42)	*P*
Age (years)	57.44 ± 8.47	58.93 ± 7.07	59.02 ± 10.12	57.76 ± 8.05	0.185
Sex (F/M)	19/26	22/22	22/23	20/22	0.887
Height (cm)	165.29 ± 7.05	165.19 ± 7.58	165.71 ± 8.89	165.50 ± 8.43	0.714
BMI (kg/m^2^)	25.28 ± 2.72	24.85 ± 3.17	24.45 ± 2.78	24.75 ± 3.40	0.583
PBW (kg)	59.31 ± 8.44	59.93 ± 8.65	59.69 ± 10.12	59.62 ± 8.70	0.888
ASA (I/II/III)	4/31/10	6/26/12	5/28/12	3/28/11	0.731
ARISCAT score					
Intermediate/high	29/16	28/16	30/15	22/20	0.527
Preoperative haemoglobin (mg/dL)	133.60 (124.80, 144.40)	137.55 (119.80, 144.40)	131.00 (123.95, 140.80)	138.20 (128.58, 145.88)	0.583
Comorbidity					
Hypertension	12 (26.7%)	22 (50.0%)	14 (31.1%)	18 (42.9%)	0.090
Diabetes	2 (4.4%)	8 (18.2%)	6 (13.3%)	4 (9.5%)	0.214
CVD	10 (22.2%)	4 (9.1%)	4 (8.9%)	6 (14.3%)	0.215
Smoking					
No/current	38/7	33/11	32/13	32/10	0.498
Creatinine (*μ*mol/L)	67.70 (60.95, 74.95)	69.25 (59.13, 80.45)	68.90 (61.70, 75.05)	69.50 (63.68, 79.43)	0.714
LEF (%)	65.00 (62.00, 70.00)	65.00 (61.00, 67.75)	66.00 (63.00, 68.00)	65.50 (61.75, 69.00)	0.720
FVC (L)	3.21 ± 0.73	3.11 ± 0.80	3.05 ± 0.71	3.34 ± 0.81	0.102
FEV_1_ (%)	98.00 (89.00, 104.00)	95.50 (82.95, 105.48)	96.00 (80.50, 104.50)	101.00 (89.00, 110.25)	0.287
FEV_1_/FVC (%)	79.84 ± 6.20	77.94 ± 8.10	78.90 ± 9.41	78.68 ± 9.50	0.632
Preoperative SaO_2_ (%)	97.30 (95.85, 97.80)	97.10 (96.20, 97.65)	96.70 (95.95, 97.55)	97.10 (96.28, 98.00)	0.795

Data are expressed as mean ± standard deviation (SD), numbers, or median (interquartile range). F: female; M: male; PBW: predicted body weight, calculated as follows: for women (45.5 + 0.91) × (height in cm − 152.4), for men (50.0 + 0.91) × (height in cm − 152.4); ASA: American Society of Anesthesiologist; BMI: body mass index; CVD: coronary vessel disease; LEF: left ventricle ejection fraction; FVC: forced vital capacity; FEV_1_: forced expiratory volume in the first second; SaO_2_: oxyhaemoglobin saturation.

**Table 2:** Intraoperative characteristics.

	PCV+OLA (*n* = 45)	PCV (*n* = 44)	VCV+OLA (*n* = 45)	VCV (*n* = 42)	*P*
Type of surgery					
Lobectomy	21 (46.7%)	24 (54.5%)	25 (55.6%)	23 (54.8%)	0.664
Wedge resection	20 (44.4%)	13 (29.5%)	13 (28.9%)	12 (28.6%)	
Segmentectomy	4 (8.9%)	7 (15.9%)	7 (15.6%)	7 (16.7%)	
Double lumen tube					
Left/right	39/6	42/2	43/2	39/3	0.331
Vasoactive drugs	25 (55.6%)	23 (52.3%)	26 (57.8%)	22 (52.4%)	0.945
Volume of fluids (mL)	1002.50 (922.50, 1158.54)	1028.75 (925.52, 1163.00)	1068.75 (902.63, 1153.65)	1057.50 (917.71, 1270.94)	0.685
Urine output (mL)	300.00 (200.00, 500.00)	275.00 (200.00, 400.00)	300.00 (200.00, 400.00)	300.00 (200.00, 500.00)	0.401
Duration of operation (min)	140.00 (100.00, 193.50)	147.50 (107.50, 189.50)	155.00 (107.50, 190.00)	170.00 (114.25, 220.50)	0.339
Duration of anesthesia (min)	180.00 (140.00, 237.50)	195.00 (141.25, 225.00)	200.00 (145.00, 228.50)	212.50 (157.50, 262.50)	0.400
Duration of OLV (min)	120.00 (87.50, 175.00)	133.00 (96.25, 170.00)	135.00 (92.50, 175.00)	160.00 (100.00, 200.00)	0.352
Blood loss (mL)	40.00 (10.00, 50.00)	40.00 (10.00, 50.00)	50.00 (10.00, 50.00)	50.00 (10.00, 100.00)	0.489
HR					
T_1_	76.16 ± 7.78	74.00 ± 8.86	74.93 ± 8.05	73.79 ± 9.36	0.548
T_2_	77.04 ± 7.07	74.23 ± 6.63	76.78 ± 8.08	77.00 ± 6.86	0.226
T_3_	76.31 ± 5.76	75.50 ± 6.18	77.20 ± 6.44	76.41 ± 5.92	0.539
MAP					
T_1_	89.44 ± 9.10	89.34 ± 8.14	91.73 ± 7.20	91.05 ± 8.16	0.422
T_2_	93.00 ± 6.54	92.09 ± 7.40	92.20 ± 7.18	92.98 ± 7.97	0.343
T_3_	91.87 ± 5.23	90.93 ± 6.52	91.69 ± 6.86	90.55 ± 7.22	0.138

Data are expressed as mean ± standard deviation (SD), numbers, or median (interquartile range). OLV: one-lung ventilation; HR: heart rate; T_1_: total-lung ventilation 10 min after induction; T_2_: one-lung ventilation 45 min; T_3_: total-lung ventilation 10 min after one-lung ventilation; MAP: mean arterial pressure.

**Table 3:** Ventilatory parameters, respiratory system mechanics, blood gas, and oxygenation parameters.

	PCV+OLA (*n* = 45)	PCV (*n* = 44)	VCV+OLA (*n* = 45)	VCV (*n* = 42)	*P*
*V*_T_ (mL)					
T_1_	432.00 (405.00, 475.00)	429.50 (404.50, 499.75)	445.00 (399.50, 502.00)	449.50 (410.25, 503.25)	0.841
T_2_	314.09 ± 34.35	324.98 ± 39.64	318.29 ± 47.47	326.90 ± 44.93	0.451
T_3_	431.00 (408.00, 500.50)	442.50 (411.50, 502.00)	452.00 (405.00, 503.00)	441.00 (407.00, 507.25)	0.936
PEEP (cmH_2_O)					
T_1_	5.00	5.00	5.00	5.00	—
T_2_	8.00 (8.00, 10.00)^∗^^▲^	5.00	10.00 (8.00, 12.00)^∗^^▲^	5.00	<0.001
T_3_	8.00 (8.00, 10.00)^∗^^▲^	5.00	10.00 (8.00, 12.00)^∗^^▲^	5.00	<0.001
*P*_peak__(_cmH_2_O)					
T_1_	18.71 ± 2.62	17.93 ± 2.98	19.16 ± 2.43	18.64 ± 3.45	0.255
T_2_	21.00 (20.00, 22.00)^△▲^	22.00 (19.25, 23.00)^△▲^	24.00 (23.00, 25.00)	23.00 (21.00, 25.00)	<0.001
T_3_	20.22 ± 2.39	18.82 ± 2.95△	21.84 ± 2.49	18.69 ± 4.69△	<0.001
*P*_mean__(_cmH_2_O)					
T_1_	10.00 (9.00, 11.00)	10.00 (9.00, 11.00)	10.00 (9.00, 11.00)	10.00 (9.00, 11.00)	0.245
T_2_	13.00 (12.00, 13.00)^∗^^▲^	11.00 (10.00, 12.00)	12.00 (12.00, 14.00)^∗^^▲^	11.00 (10.00, 12.00)	<0.001
T_3_	12.00 (11.00, 13.00)^∗^^▲^	11.00 (10.00, 12.00)	12.00 (11.00, 13.00)^∗^^▲^	10.00 (8.00, 11.00)	<0.001
Cdyn (mL/cmH_2_O)					
T_1_	46.60 ± 5.60	46.36 ± 6.57	45.78 ± 7.51	46.17 ± 6.25	0.943
T_2_	27.00 (24.00, 32.00)^∗^^▲^	23.00 (21.00, 25.00)	27.00 (22.00, 30.00)^∗^^▲^	20.00 (18.75, 21.00)	<0.001
T_3_	47.33 ± 5.59^∗^^▲^	44.78 ± 4.25^▲^	46.18 ± 5.22^▲^	43.12 ± 5.20	0.001
PaCO_2_ (mmHg)					
T_1_	42.29 ± 3.76	43.30 ± 3.76	43.78 ± 4.38	43.57 ± 4.77	0.345
T_2_	44.43 ± 3.90	44.17 ± 4.89	45.26 ± 4.71	44.20 ± 4.81	0.485
T_3_	43.00 (39.50, 47.00)	45.00 (41.00, 47.00)	46.00 (41.00, 48.00)	44.00 (41.00, 47.25)	0.455
pH					
T_1_	7.40 ± 0.03	7.39 ± 0.03	7.39 ± 0.04	7.39 ± 0.04	0.402
T_2_	7.38 (7.35, 7.40)	7.38 (7.35, 7.42)	7.37 (7.39, 7.40)	7.38 (7.35, 7.41)	0.633
T_3_	7.38 ± 0.04	7.38 ± 0.03	7.38 ± 0.05	7.38 ± 0.04	0.796
*V*_D_/*V*_T_					
T_1_	0.17 ± 0.04	0.18 ± 0.06	0.19 ± 0.06	0.19 ± 0.05	0.210
T_2_	0.18 ± 0.05^∗^^▲^	0.21 ± 0.07	0.19 ± 0.07^∗^^▲^	0.22 ± 0.06	0.003
T_3_	0.16 (0.13, 0.21)^∗^^▲^	0.20 (0.17, 0.24)	0.19 (0.14, 0.22)	0.21 (0.17, 0.24)	<0.001
Qs/Qt					
T_1_	0.16 ± 0.03	0.16 ± 0.03	0.16 ± 0.04	0.16 ± 0.03	0.873
T_2_	0.17 (0.16, 0.19)^∗^^▲^	0.19 (0.18, 0.20)	0.18 (0.17, 0.19)	0.19 (0.17, 0.20)	0.006
T_3_	0.17 ± 0.03	0.17 ± 0.03	0.17 ± 0.03	0.18 ± 0.03	0.280
PaO_2_/FiO_2_ ratio					
T_1_	341.91 ± 78.37	350.95 ± 62.70	342.47 ± 81.27	352.10 ± 74.73	0.875
T_2_	173.75 (138.13, 221.87)^▲^	153.13 (109.38, 185.94)	166.25 (146.25, 200.63)^▲^	134.38 (106.25, 180.63)	0.002
T_3_	330.69 ± 65.24	327.40 ± 64.31	317.60 ± 65.71	304.12 ± 73.40	0.264

Data are expressed as mean ± standard deviation (SD) or median (interquartile range). *V*_T_: tidal volume; T_1_: total-lung ventilation 10 min after induction; T_2_: one-lung ventilation 45 min; T_3_: total-lung ventilation 10 min after one-lung ventilation; PEEP: positive end-expiratory pressure; *P*_peak_: peak airway pressure; *P*_mean_: mean airway pressure; Cdyn: dynamic compliance; PaCO_2_: partial pressure of arterial carbon dioxide; *V*_D_/*V*_T_: dead space fraction; Qs/Qt: intrapulmonary shunt ratio; PaO_2_/FiO_2_: arterial partial pressure of oxygen/fraction of inspired oxygen. ^∗^*P* < 0.05 versus PCV, ^△^*P* < 0.05 versus VCV+OLA, and ^▲^*P* < 0.05 versus VCV.

**Table 4:** Other clinical endpoints.

	PCV+OLA (*n* = 45)	PCV (*n* = 44)	VCV+OLA (*n* = 45)	VCV (*n* = 42)	*P*
Pneumonia	2 (4.4%)	5 (11.4%)	4 (8.9%)	7 (16.7%)	0.312
Atelectasis	1 (2.2%)	4 (9.1%)	2 (4.4%)	6 (14.3%)	0.148
Acute respiratory failure	0 (0%)	1 (2.3%)	1 (2.2%)	4 (9.5%)	0.096
The duration of ICU stay (hours)	32.00 (25.00, 37.00)^∗^^△▲^	39.75 (32.88, 43.00)	39.50 (27.00, 43.50)	39.60 (24.88, 43.70)	<0.001
The duration of hospital stay after surgery (days)	6.00 (5.00, 7.00)	6.00 (5.00, 8.00)	6.00 (5.00, 8.00)	6.00 (4.75, 7.00)	0.204

Data are expressed as median (interquartile range) or numbers. ICU: intensive care unit. ^∗^*P* < 0.05 versus PCV, ^△^*P* < 0.05 versus VCV+OLA, and ^▲^*P* < 0.05 versus VCV.

## Data Availability

The clinical data used to support the findings of this study are available from the corresponding author upon reasonable request.
